# Expanding the phenome and variome of the ROBO-SLIT pathway in congenital heart defects: toward improving the genetic testing yield of CHD

**DOI:** 10.1186/s12967-023-03994-y

**Published:** 2023-02-28

**Authors:** Hager Jaouadi, Chris Jopling, Fanny Bajolle, Alexis Théron, Adèle Faucherre, Hilla Gerard, Sarab Al Dybiat, Caroline Ovaert, Damien Bonnet, Jean-François Avierinos, Stéphane Zaffran

**Affiliations:** 1 Marseille Medical Genetics (MMG) U1251, Aix Marseille Université, INSERM, 13005 Marseille, France; 2 Institute of Functional Genomics (IGF), University of Montpellier, CNRS, INSERM, LabEx ICST, Montpellier, France; 3 Service de Cardiologie Congénitale Et Pédiatrique, Centre de Référence Malformations Cardiaques Congénitales Complexes - M3C, Hôpital Necker-Enfants Malades, APHP and Université Paris Cité, Paris, France; 4 Department of Cardiac Surgery, La Timone Hospital, AP-HM, Marseille, France; 5 Department of Cardiology, La Timone Hospital, AP-HM, Marseille, France; 6 Department of Pediatric Cardiology, Timone Enfant Hospital, AP-HM, Marseille, France

**Keywords:** Congenital heart defects, Robo-Slit pathway, Genetics, Exome sequencing

## Abstract

**Background:**

Recent studies have shown the implication of the ROBO-SLIT pathway in heart development. Within this study, we aimed to further assess the implication of the *ROBO* and *SLIT* genes mainly in bicuspid aortic valve (BAV) and other human congenital heart defects (CHD).

**Methods:**

We have analyzed a cohort of singleton exome sequencing data comprising 40 adult BAV patients, 20 pediatric BAV patients generated by the Pediatric Cardiac Genomics Consortium, 10 pediatric cases with tetralogy of Fallot (ToF), and one case with coarctation of the aorta. A gene-centered analysis of data was performed. To further advance the interpretation of the variants, we intended to combine more than 5 prediction tools comprising the assessment of protein structure and stability.

**Results:**

A total of 24 variants were identified. Only 4 adult BAV patients (10%) had missense variants in the *ROBO* and *SLIT* genes. In contrast, 19 pediatric cases carried variants in *ROBO* or *SLIT* genes (61%). Three BAV patients with a severe phenotype were digenic. Segregation analysis was possible for two BAV patients. For the homozygous *ROBO4*: p.(Arg776Cys) variant, family segregation was consistent with an autosomal recessive pattern of inheritance. The *ROBO4*: c.3001 + 3G > A variant segregates with the affected family members. Interestingly, these variants were also found in two unrelated patients with ToF highlighting that the same variant in the *ROBO4* gene may underlie different cardiac phenotypes affecting the outflow tract development.

**Conclusion:**

Our results further reinforce the implication of the *ROBO4* gene not only in BAV but also in ToF hence the importance of its inclusion in clinical genetic testing. The remaining *ROBO* and *SLIT* genes may be screened in patients with negative or inconclusive genetic tests.

**Supplementary Information:**

The online version contains supplementary material available at 10.1186/s12967-023-03994-y.

## Introduction

The secreted SLIT glycoproteins and their Roundabout (ROBO) receptors were initially known for axon guidance and dendritic branching in the developing central nervous system [1–3]. Subsequently, several studies have expanded the functional spectrum of the ROBO-SLIT pathway by reporting other functions, such as cell migration and proliferation, angiogenesis, and vascularization in different organs and tissues [3–5]. Recently, a pivotal role of the SLIT ligands and their ROBO receptors has been reported in animal heart morphogenesis and development [6–8]. These findings have been reinforced by the identification of genetic variations in *ROBO1* and *ROBO4* genes in patients with tetralogy of Fallot (ToF) and bicuspid aortic valve (BAV) disease, respectively [9, 10]. Indeed, Kruszka et al. identified loss of function variants in *ROBO1* gene in three unrelated patients with ToF and ventricular septal defects (VSD) [9]. More recently, Jaouadi et al. identified a *ROBO1* variant in a BAV family with three affected members [11].

In 2019, Gould et al. reported variants in the *ROBO4* gene in patients with BAV and ascending aortic aneurysms (AscAA). The phenotypes observed in *Robo4* animal models were consistent with patients’ phenotypes with a novel endothelial etiology supporting a causative role of *ROBO4* [10]. Thereafter, additional variants in *ROBO4* have been linked to human BAV [12]. The authors have concluded that variants in *ROBO4* along with *NOTCH1*, *GATA4* and *SMAD6* are enriched in BAV-patients with early onset complications [12].

Albeit human genetic variations have been identified in *ROBO* genes, mainly *ROBO4* and *ROBO1*, data from several animal models point out the implication of the remaining *ROBO* and *SLIT* genes in CHD pathogenesis [6, 13, 14]. Moreover, Zhao et al. (2022) have underlined the clinical relevance of *SLIT3* as a promising candidate gene for further screening in patients [13].

In the present study, we aimed firstly to screen adult and pediatric patients with BAV in order to identify genetic variants in *ROBO* and *SLIT* genes using exome sequencing data combined to a thorough in silico analysis. Based on the results of this analysis, we sought to expand the pediatric cohort to include other CHD phenotypes (10 patients with ToF and one case with coarctation of the aorta (CoA)) in order to determine whether variants in *ROBO* and *SLIT* genes may be implicated in CHD other than BAV.

Of note, the study includes CHD patients with no relevant variants in known CHD-related genes such as *NOTCH1*, *NOTCH2*, *GATA5*, *GATA4*, *ACTA2*, *SMAD6*, *NKX2-5*, *FLT4*, *TGFBR1*, and *TGFBR2*.

## Patients and methods

This study was performed according to the principles of the Declaration of Helsinki and to the ethical standards of the first author’s institutional review board. The patients provided their written informed consent to participate in this study (approved by the Marseille ethic committee n°13.061 and 2016-A00958-53). Personal health data and DNA from two pediatric BAV patients and their related are part of the CARREG study (http://carreg.fr/en/), which was declared to the French national committee for informatics and liberties (France; CNIL; No. 1734573V0). The CARREG study is a prospective monocenter study promoted by the “Centre de Référence des Malformations Cardiaques Congénitales Complexes (M3C)” located at the Pediatric cardiology department of the Necker-Enfants Malades Hospital, Paris, France. Clinical records were reviewed by cardiologist or pediatric-cardiologist before recruitment and cardiovascular diagnosis was obtained by echocardiography mainly. Patients with 22q11.2 deletion or other recognized syndromes were excluded.

### Patients

The starting study cohort includes a total of 71 patients with clinical diagnosis of bicuspid aortic valve (40 adult and 20 pediatric patients), tetralogy of Fallot (10 pediatric cases) and one pediatric case with coarctation of the aorta (CoA). No other defects are associated with the main clinical diagnosis with confirmed absence of structural myocardial and syndromic diseases.

### Exome sequencing

Germline DNA was extracted from blood samples and subjected to exome sequencing. Whole exome sequencing (WES) was performed by the Genomics and Bioinformatics Platform (GBiM) of the INSERM U1251 Marseille Medical Genetics facility using the NimbleGen SeqCap EZ MedExome kit (total design size 47 Mb) according to the manufacturer’s protocol (Roche Sequencing Solutions, Madison, USA). All DNA and libraries preparations (KAPA HyperPrep Kits (Roche)) were performed according to the manufacturers’ instructions. The DNA libraries were subjected to paired-end sequencing using the Illumina NextSeq500 sequencing platform (Illumina, San Diego, CA, USA). Raw fastQ files were aligned to the hg19 reference human genome (University of California Santa Cruz, UCSC) using BWA software [15]. Variant calling workflow was performed according to the GATK best practices [16]. Both HaplotypeCaller and BaseRecalibration tools have been used for variant calling and quality score recalibration. The output files were annotated using ANNOVAR software [17]. On average, a depth of 125X and a coverage of 97.7% of the bases at 30X have been obtained per sample.

#### Variant annotation and prioritization

Variant annotation process and exome data analysis were performed using VarAFT software version 2.17–2 (http://varaft.eu/) [18]. Firstly, a patient- centered approach was applied. Thus, we excluded variants with a minor allele frequency (MAF) > 1% in gnomAD (Genome Aggregation Database) (http://gnomad.broadinstitute.org/). Then, we removed non-coding and synonymous variants with no impact on splicing with HSF-Pro tool. Subsequently, the remaining variants were filtered based on their in silico pathogenicity prediction with UMD_Predictor, SIFT and PolyPhen tools [19–21]. The prioritized variants were finally interpreted according to their clinical relevance. Indeed, patients with likely pathogenic and/or causative variants in genes linked to the NOTCH or TGFβ pathways or in cardiac transcription factors such as *GATA4/5*, *NKX2-5*, and *TBX-5* have been selected for further analysis and excluded from the present study.

As a second step, patients with no-relevant variants in CHD-related genes were re-analyzed as following: a gene-centered approach was applied to the remaining patients toward identifying variants in the ROBO-SLIT pathway. Thus, we used a gene list including *ROBO1*, *ROBO2*, *ROBO3*, *ROBO4*, *SLIT1*, *SLIT2*, and *SLIT3* genes to run the same prioritization strategy as above. The main functions of *ROBO* and *SLIT* genes are summarized in Table 1.

**Table 1:** The main functions of *ROBO* and *SLIT* genes

Gene	Gene_name	HGNC ID	GeneRIF: Gene Reference into Function from NCBI
*ROBO1*	Roundabout guidance receptor 1	HGNC:10249	Aortic valve development, axon guidance, axonogenesis, brain and heart development
*ROBO2*	Roundabout guidance receptor 2	HGNC:10250	Aortic valve development, apoptotic process involved in development, axon guidance, axonogenesis, brain and heart development, female gonad development, female sex differentiation
*ROBO3*	Roundabout guidance receptor 3	HGNC:13433	Axon guidance, axonogenesis, cell recognition, cell–cell adhesion via plasma-membrane adhesion molecules, neuron projection guidance, neuron recognition
*ROBO4*	Roundabout guidance receptor 4	HGNC:17985	Axonogenesis, cell recognition, cell–cell adhesion via plasma-membrane adhesion molecules, neuron projection guidance, neuron recognition
*SLIT1*	Slit guidance ligand 1	HGNC:11085	Axon extension, axon guidance, axonogenesis, brain development, central nervous system neuron development
*SLIT2*	Slit guidance ligand 2	HGNC:11086	Actin filament polymerization, aortic valve development, apoptotic process involved in development, axon extension, axon guidance, axonogene sis, brain development
*SLIT3*	Slit guidance ligand 3	HGNC:11087	Aortic valve development, apoptotic process involved in development, axon extension, axon guidance, axonogenesis, brain development, cardiac chamber development and morphogenesis

Source: https://www.genenames.org/

#### Combined Annotation Dependent Depletion (CADD)

Given the lack of detailed clinical description for some patients and the family history that would allow for segregation analysis, we used the CADD computational algorithm to further assess variants pathogenicity.

For annotation, CADD used the Ensembl Variant Effect Predictor, data from the ENCODE project and information from UCSC genome browser tracks. These annotations span a wide range of data types including conservation metrics such as GERP, phastCons, and phyloP; functional genomic data like DNase hypersensitivity and transcription factor binding; transcript information like distance to exon–intron boundaries or expression levels in commonly studied cell lines; and protein-level scores like Grantham, SIFT, and PolyPhen [22, 23]. Thus, CADD algorithm simulate neutral and deleterious variants from multiple species alignments, annotate variants based on the conservation among species, genetic context and epigenetics, rank the variants by a logistic regression model and finally generate a CADD score for each variant in the human genome.

A scaled C-score of greater of equal 10 indicates that these are predicted to be the 10% most deleterious substitutions that you can do to the human genome, a score of greater or equal 20 indicates the 1% most deleterious and so on.

To identify potentially pathogenic variants, a cutoff between 10 and 20 can be set. A cutoff of 15 is recommended as it is the median value for all possible canonical splice site changes and non-synonymous variants in CADD v1.0.

### In silico assessment of protein stability and interactions

#### I-mutant

In order to aid the annotation process, an in silico prediction of protein stability free energy change (DDG) was performed using I-Mutant3.0 software (http://gpcr.biocomp.unibo.it/cgi/predictors/I-Mutant3.0/I-Mutant3.0.cgi) [24]. The substitutions are ranked according to a three-state classification system: destabilizing mutations (DDG < − 0.5 kcal/mol), stabilizing mutations (DDG > 0.5 kcal/mol) and neutral mutations (− 0.5 <  = DDG <  = 0.5 kcal/mol).

#### Project HOPE

The project HOPE tool (https://www3.cmbi.umcn.nl/hope) is a web service that analyses the structural and physicochemical effects of point mutations in a protein sequence using PDB file when the corresponding protein structure has been solved experimentally (95–100% match). Whenever this is not the case, HOPE will build a homology model using an existing template (between 30 and 95% match). As an estimation, HOPE uses information obtained from the 3D-structure in 60–70% of the cases [25].

## Results

From a cohort of 40 BAV adult patients [26] and 20 BAV pediatric cases, we sought to determine the implication of the ROBO-SLIT pathway in patients with no relevant variants in known BAV-related genes. Interestingly, the yield of rare variants in *ROBO* and *SLIT* genes was greater in the pediatric cohort (13/20, 65%) compared to only 4 out 40 BAV cases (10%) from the adult cohort (Table 2).

**Table 2:** List of the identified variants and patients’ phenotypes

Gene	HGVSc	HGVSp	Patient phenotype	Patient ID		Sex	
*ROBO1*	c.1828G > A	p.Val610Ile	BAV-adult	BAV-AD-1		M	
*ROBO2*	c.639C > G	p.Asp213Glu	BAV-adult	BAV-AD-2		F	
*ROBO2*	c.2431C > T	p.Arg811Trp	BAV-Ped	BAV-PED-1		M	
*ROBO3*	c.968C > T	p.Thr323Met	ToF	ToF-PED-2		M	
*ROBO3*	c.1615C > T	p.Arg539Trp	ToF	ToF-PED-3		F	
*ROBO3*	c.2576C > A	p.Pro859Gln	BAV-Ped	BAV-PED-4		M	
*ROBO3*	c.2993G > T	p.Gly998Val	CoA-Ped	CoA-PED-5		M	
*ROBO3*	c.3478C > T	p.Pro1160Ser	ToF	ToF-PED-6		F	
*ROBO4*	c.908C > A	p.Ala303Asp	BAV-Ped	BAV-PED-7		F	
*ROBO4*	c.1337C > A	p.Ala446Asp	BAV-Ped	BAV-PED-8		M	
*ROBO4*	c.2326C > T	p.Arg776Cys	ToF / BAV-Ped (unrelated patients)	ToF-PED-9	BAV-PED-10	F	F
*ROBO4*	c.2723G > A	p.Arg908Gln	BAV-Ped	BAV-PED-1		M	
*ROBO4*	c.3001 + 3G > A	–	ToF / BAV-Ped (unrelated patients)	ToF-PED-11	BAV-PED-12	M	M
*SLIT1*	c.446C > T	p.Pro149Leu	BAV-Ped	BAV-PED-13		M	
*SLIT1*	c.789C > A	p.Cys263Ter	BAV-Ped	BAV-PED-14		F	
*SLIT1*	c.1363C > A	p.Arg455Ser	BAV-Ped	BAV-PED-15		M	
*SLIT1*	c.3757G > A	p.Ala1253Thr	BAV-Ped	BAV-PED-16		M	
*SLIT1*	c.4020A > C	p.Glu1340Asp	BAV-Ped	BAV-PED-7		F	
*SLIT1*	c.4145A > G	p.His1382Arg	BAV-adult	BAV-AD-3		F	
*SLIT1*	c.4202G > T	p.Cys1401Phe	BAV-Ped	BAV-PED-17		M	
*SLIT2*	c.3877C > A	p.Leu1293Met	BAV-Ped	BAV-PED-13		M	
*SLIT3*	c.1481G > C	p.Arg494Thr	BAV-Ped	BAV-PED-18		F	
*SLIT3*	c.1886G > A	p.Ser629Asn	BAV-adult	BAV-AD-4		F	
*SLIT3*	c.4086C > A	p.Cys1355Ter	BAV-Ped	BAV-PED-19		M	

Family segregation was performed for two BAV patients only, both with *ROBO4* variants.

The first patient (BAV-PED-10) is a male pediatric case with BAV. His medical records include small aortic insufficiency in the posterior commissure, fusion of the anterior commissure, right anterior leaflet prolapse, aortic annulus dilatation and dilated ascending aorta (z score 3.2 and 3.3). The patient had a positive family history of aortic valve defects. His paternal and maternal grand-mothers underwent aortic valve replacement.

**Fig. 1:**
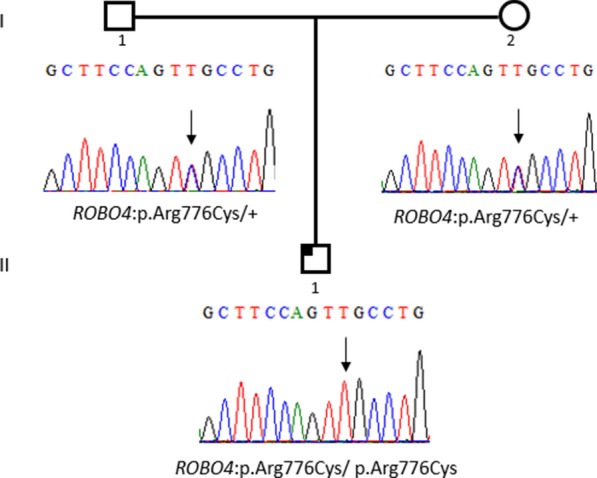
Family segregation of the *ROBO4*: p.Arg776Cys variant. Darkened left upper quadrant: Affected child with BAV

The patient (BAV-PED-10) carried a homozygous *ROBO4* variant (p. Arg776Cys) (Table 3). His parents were found heterozygous for the variant (Fig. 1). The MAF of this variant (rs138481093) is 0.004699 in gnomAD with a total number of homozygotes equal to 8. Of note, our patient is of white European non-Finnish ethnic group which represents the highest MAF population (0.007781).

**Table 3:** Variants coordinates

Location	Gene	RefSeq Match	cDNA_ position	CDS_ position	Protein_ position	Amino_ acids	Codons	Read depth	Exon	Variant type	gnomAD_ Allele Freq	Rs_number
3:78717171-78717171	*ROBO1*	NM_002941	1942	1828	610	V/I	Gta/Ata	84	14	Missense	0.0005077	rs141178745
3:77530342-77530342	*ROBO2*	NM_002942	695	639	213	D/E	gaC/gaG	101	4	Missense	0.0003215	rs184080216
3:77629200-77629200	*ROBO2*	NM_002942	3331	2431	811	R/W	Cgg/Tgg	200	16	Missense	0.008396	rs188582283
11:124,740,559–124,740,559	*ROBO3*	NM_022370	1160	968	323	T/M	aCg/aTg	55	6	Missense	0.002300	rs151168595
11:124743284-124743284	*ROBO3*	NM_022370	1807	1615	539	R/W	Cgg/Tgg	171	10	Missense	0.003234	rs139930558
11:124746004-124746004	*ROBO3*	NM_022370	2768	2576	859	P/Q	cCa/cAa	55	16	Missense	–	–
11:124747839-124747839	*ROBO3*	NM_022370	3185	2993	998	G/V	gGa/gTa	40	21	Missense	0.001755	rs75098003
11:124748637-124748637	*ROBO3*	NM_022370	3670	3478	1160	P/S	Cct/Tct	259	23	Missense	–	–
11:124765481-124765481	*ROBO4*	NM_019055	1394	908	303	A/D	cGg/cTg	71	6	Missense	–	–
11:124763923-124763923	*ROBO4*	NM_019055	1823	1337	446	A/D	cGg/cTg	43	9	Missense	0.000	rs1287612263
11:124756982-124756982	*ROBO4*	NM_019055	2812	2326	776	R/C	Gcg/Acg	96	15	Missense	0.004699	rs138481093
11:124756431-124756431	*ROBO4*	NM_019055	3209	2723	908	R/Q	gCc/gTc	77	16	Missense	3.891e-05	rs747627515
11:124754934-124754934	*ROBO4*	NM_019055	-	-	-	-	-	270	-	Splice_donor_region	0.009486	rs145918924
10:98825811-98825811	*SLIT1*	NM_003061	692	446	149	P/L	gGg/gAg	257	5	Missense	0.000	rs1459814303
10:98823216-98823216	*SLIT1*	NM_003061	1035	789	263	C/*	acG/acT	67	8	Stop_gained	**–**	**–**
10:98808814-98808814	*SLIT1*	NM_003061	1609	1363	455	R/S	Gcg/Tcg	72	14	Missense	–	–
10:98763933-98763933	*SLIT1*	NM_003061	4003	3757	1253	A/T	Cgg/Tgg	49	34	Missense	8.131e-05	rs751020526
10:98762595-98762595	*SLIT1*	NM_003061	4266	4020	1340	E/D	ctT/ctG	70	35	Missense	0.002267	rs747965419
10:98762470-98762470	*SLIT1*	NM_003061	4391	4145	1382	H/R	cAt/cGt	539	35	Missense	1.773e-05	rs768287574
10:98762079-98762079	*SLIT1*	NM_003061	4448	4202	1401	C/F	aCg/aAg	52	36	Missense	–	–
4:20618562-20618562	*SLIT2*	NM_004787	4129	3877	1293	L/M	Ctg/Atg	67	35	Missense	–	–
5:168189673-168189673	*SLIT3*	NM_003062	1901	1481	494	R/T	tCc/tGc	85	15	Missense	0.0009129	rs147560182
5:168180047-168180047	*SLIT3*	NM_003062	2306	1886	629	S/N	tCa/tTa	144	18	Missense	0.007048	rs34260167
5:168098265-168098265	*SLIT3*	NM_003062	4485	4065	1355	C/*	acG/acT	88	34	Stop_gained	–	–

Last check of allele frequencies was performed using gnomAD browser on 24 November 2022

The second case (BAV-PED-12) is a male pediatric case with BAV. The analysis of his WES data allowed us to identify a splice site heterozygous variant in *ROBO4* (c.3001 + 3G > A). The patient’s father was found to have hypoplastic left coronary artery and his brother had VSD. His mother and sister are healthy.

The *ROBO4*: c.(3001 + 3G > A) variant was found in the patient’s father (I-1) and brother (II-2). The mother (I-2) and sister (II-3) do not carry the variant.

**Fig. 2:**
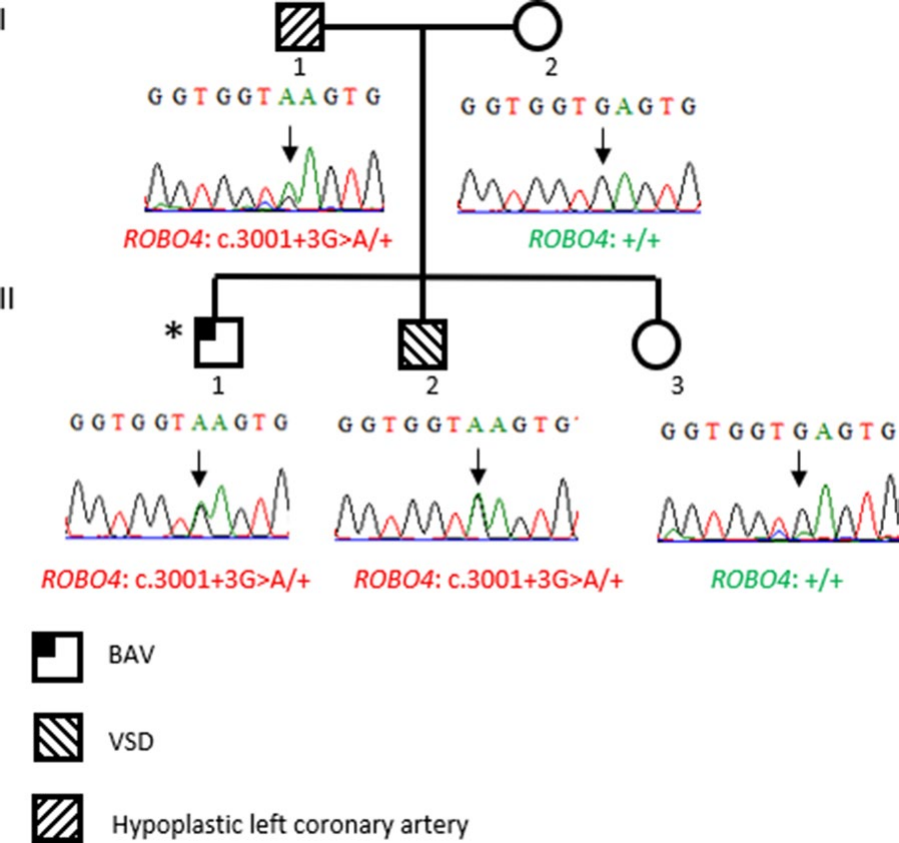
Family segregation of the *ROBO4*: c.3001 + 3G > A variant. The index-case (BAV-PED-12) is marked with a star

Family pedigree and segregation are shown in Fig. 2.

As for the first patient (BAV-PED-10), this case is European non-Finnish also. The MAF of the *ROBO4*: c.3001 + 3G > A variant in this population is 0.01448. The highest population MAF of this variant (rs145918924) is 0.02831 in the Ashkenazi Jewish population.

Of note, Gould et al. reported a heterozygous splice site variant *ROBO4*: c.2056 + 1G > T in a multigenerational BAV-family. Interestingly, seven of eight affected cases were male [10]. These findings underline the intrafamilial variability as well as the phenotypic pleiotropy of *ROBO4* variants.

As we mentioned above, the identification of more *ROBO* and *SLIT* variants in the pediatric BAV cases than in the adult cohort prompted us to investigate the implication of this pathway in another CHD phenotype. Thus, we analyzed 10 ToF patients and one CoA case. Five out 10 ToF patients carried variants in *ROBO* and *SLIT* genes. Three patients carried variants in the *ROBO3* gene and strikingly, the two other patients (ToF-PED-9 and ToF-PED11) were carrying the aforementioned *ROBO4* variants (p.(Arg776Cys) and c.3001 + 3G > A) at a heterozygous state (Table 2).

The clinical resume of the ToF-patient (ToF-PED-9) carrying the *ROBO4*: p.Arg776Cys variant is as following: Pregnancy was complicated by gestational diabetes. Pulmonary atresia and VSD as well as partial corpus callosum agenesis were prenatally diagnosed. Amniocentesis was refused by the parents. The anatomy was confirmed after birth. Pulmonary arteries were noted to be extremely hypoplastic (2 mm, z-value -5, birth weight 4 kg). A malformation of the arterial duct was noted, with no signs of spontaneous closure. At the age of 27 months, a total cardiac repair was performed.

The clinical resume of the ToF-patient (ToF-PED-11) carrying the *ROBO4*: c.3001 + 3G > A splicing variant is as following: Severe ToF and thoraco-abdominal situs inversus was prenatally diagnosed. Birth weight was small for gestational age (2.4 kg, 38W). Anatomy was confirmed after birth. Pulmonary annulus was very hypoplastic (Z value − 3.7) as well as pulmonary arteries (Z value RPA − 2.4, LPA − 2). The complete cardiac repair (closure VSD and patch enlargement of pulmonary valve and artery) was performed 11 months later.

ToF is defined by the presence of four cardiac defects namely; ventricular septal defect (VSD), pulmonary valve stenosis, right ventricular hypertrophy and overriding aorta, which potentially arise from a misalignment of the great arteries [27, 28]. The identification of the same *ROBO4* variants in BAV and ToF patients points out the pleiotropic role of this gene with its implication in several CHD entities with different pattern of inheritance. This pleiotropy can be explained by the potential contribution of *ROBO4* gene in different cardiac cell populations [8, 13, 29], but also by the difference of the genetic background of each individual and epigenetics mechanisms acting during heart morphogenesis.

Three additional *ROBO4* variants were identified in the present study. The *ROBO4* variant (p. Arg908Gln) was identified in a BAV patient with aortic stenosis (BAV-PED-1). This patient carried a second missense variant in *ROBO2* gene (p. Arg811Trp). The *ROBO4*: p.(Ala303Asp) has been identified in a pediatric BAV-case (BAV-PED-7) with aneurysm. Similarly, this patient carried a second variant in the *SLIT1* gene (p.Glu1340Asp). The third *ROBO4*: p.(Ala446Asp) variant was found in BAV-PED-8 case (Table 2). No BAV-related complications were noted for this patient.

In regards to BAV adult patients with variants in *ROBO1*, *ROBO2*, *SLIT1* and *SLIT3* genes, the presence of BAV-related complications such as aortic regurgitation, aortic stenosis, and AscAA was checked. Only the patient (BAV-AD-1) with the *ROBO1*: p.(Val610Ile) variant had AscAA.

Within this study, we report two stop-gain variants in *SLIT1* (p.Cys263Ter) and *SLIT3* (p.Cys1355Ter) genes. The patient carrying the *SLIT3* stop-gain variant had BAV with mitral regurgitation.

Collectively, a total of 24 rare variants were identified including 21 missense, 2 stop-gain, and 1 splice site variants (Table 2). The majority of variants were found in the pediatric cohort. Indeed, 19 pediatric cases carried variants in *ROBO* and *SLIT* genes (19/31 CHD-patients; 61%), whereas, only 4 adult patients (10%) had missense variants in *ROBO1*, *ROBO2*, *SLIT1* and *SLIT3* genes.

It should be noted that, all the patients carried heterozygous variants except a BAV- patient (BAV-PED-10) with the homozygous *ROBO4* variant (p. Arg776Cys).

Overall, the in-silico predictions of variant pathogenicity are quite consistent among the different software tools, specifically, variants in *ROBO1*, *ROBO3*, and *SLIT* genes, were predicted to have a large decrease of protein stability and high CADD scores (Table 4). Indeed, except for *SLIT1* (p.Cys263Ter) and *SLIT3* (p.Cys1355Ter) stop-gain variant with a very high CADD-scores (36 and 42, respectively) which is mainly due to the truncating type of the variants, the highest scores (≥ 30) are attributed to variants located in the fibronectin type III-3 domain of *ROBO* genes. As an example, the *ROBO2*: p. (Arg811Trp) and the *ROBO3*: p.(Pro859Gln) variants, with CADD-scores 31 and 32, respectively, are located within the Fibronectin type-III 3 domain of each gene (Additional file 1).

**Table 4:** In silico prediction of variants pathogenicity

Variant prediction					I-Mutant 3.0	
Variant	CADD_PHRED	SIFT	PolyPhen	UMD_Prediction	DDG Value prediction	SVM3 prediction effect
*ROBO1*: p.Val610Ile	25.3	Deleterious	Probably_damaging	Probably pathogenic	− 0.07 kcal/mol	Large decrease
*ROBO2*: p.Asp213Glu	20.7	Tolerated	Benign	Probably pathogenic	− 0.24 kcal/mol	Large increase
*ROBO2:* p.Arg811Trp	31	Deleterious	Probably_damaging	Pathogenic	− 0.31 kcal/mol	Neutral
*ROBO3:* p.Thr323Met	25.4	Deleterious	Probably_damaging	Pathogenic	− 1.73 kcal/mol	Large decrease
*ROBO3:* p.Arg539Trp	23.7	Deleterious	Probably_damaging	Pathogenic	− 0.03 kcal/mol	Neutral
*ROBO3:* p.Pro859Gln	32	Deleterious	Probably_damaging	Pathogenic	− 1.33 kcal/mol	Large decrease
*ROBO3:* p.Gly998Val	24.8	Deleterious	Benign	Pathogenic	− 0.46 kcal/mol	Large decrease
*ROBO3:* p.Pro1160Ser	26.1	Deleterious	Probably_damaging	Pathogenic	− 1.06 kcal/mol	Large decrease
*ROBO4:* p.Ala303Asp	14.37	Tolerated	Benign	Pathogenic	− 0,52 kcal/mol	Neutral
*ROBO4:* p.Ala446Asp	23	Deleterious	Benign	Probably pathogenic	− 0.61 kcal/mol	Large decrease
*ROBO4:* p.Arg776Cys	22.9	Tolerated	Benign	Probably pathogenic	− 0.59 kcal/mol	Neutral
*ROBO4:* p.Arg908Gln	16.15	Tolerated	Benign	Pathogenic	− 0.67 kcal/mol	Neutral
*ROBO4:* c.3001 + 3G > A	–	–	–	–	–	–
*SLIT1:* p.Pro149Leu	24.7	Deleterious	Probably_damaging	Pathogenic	− 0.30 kcal/mol	Neutral
*SLIT1:* p.Cys263Ter	36	–	–	Pathogenic	–	–
*SLIT1:* p.Arg455Ser	27.1	Deleterious	Probably_damaging	Pathogenic	− 1.30 kcal/mol	Large decrease
*SLIT1:* p.Ala1253Thr	19.47	Tolerated	Benign	Probably pathogenic	− 0.78 kcal/mol	Large decrease
*SLIT1:* p.Glu1340Asp	15.21	Tolerated	Benign	Probably pathogenic	− 0.29 kcal/mol	Large decrease
*SLIT1:* p.His1382Arg	14.47	Tolerated	Benign	Probably pathogenic	0.11 kcal/mol	Neutral
*SLIT1:* p.Cys1401Phe	29.9	Deleterious	Probably_damaging	Pathogenic	− 0.35 kcal/mol	Large decrease
*SLIT2:* p.Leu1293Met	24.8	Deleterious	Probably_damaging	Probably pathogenic	− 1.17 kcal/mol	Large decrease
*SLIT3:* p.Arg494Thr	23.3	Deleterious	Probably_damaging	Pathogenic	− 1.10 kcal/mol	Large decrease
*SLIT3:* p.Ser629Asn	21.8	Tolerated	Benign	Pathogenic	− 0.75 kcal/mol	Neutral
*SLIT3:* p.Cys1355Ter	42	–	–	Pathogenic	–	–

A more detailed description of variant localization and their predicted impact on protein structure, interaction and physicochemical properties is provided in the Additional file 1. Sanger confirmation of the prioritized variants is provided in Additional file 2.

## Discussion

ROBO receptors and their SLIT ligands play versatile roles during heart development across species and have been associated with congenital cardiac defects (CHD) in humans [3, 7, 30]. With the exception of the mammalian ROBO4 receptor, the extracellular domain of ROBO contains 5 Ig-like domains and 3 fibronectin repeats [3, 31]. SLIT are the main ligands of ROBO receptors, which bind through their LRR2 domain to the first Ig domain of ROBO proteins [3]. Of note, SLIT ligands bind also to a wide range of extracellular matrix molecules such as type IV collagens. On the other hand heparin sulfate proteoglycans binds to both SLIT and ROBO [3]. Moreover, ROBO and SLIT proteins are involved in heart tube development of Dosophila and zebrafish and in neural crest migration and adhesion in mice. The absence of ROBO1 receptor has been linked to septal and outflow tract defects [7, 29, 32]. The knockdown of *Robo1* in zebrafish resulted in an inhibition of endocardial and myocardial migration leading to an unfused heart fields [7, 33].

In vertebrates, ROBO4 is selectively expressed in endothelial cells and plays a key role in angiogenesis and blood vessel permeability [34]. Similarly, ROBO1/2 receptors and SLIT are also expressed in endothelial cells and contribute to cell motility and polarity [35]. Functional studies have suggested that *ROBO4* mutations disrupt endothelial cells performance and impair barrier function leading to abnormal aorta remodeling [10]. Furthermore, *Robo4* knockout mice showed severe cardiovascular defects such as aortic valve thickening combined with, in some cases, BAV, aortic regurgitation, aortic stenosis and AscAA [10].

It has been shown that the SLIT-ROBO pathway is involved in the guidance of cranial neural crest cell migration [36]. Additionally, SLIT-ROBO signaling is crucial for organizing neural crest cells and placode derived neurons to form ganglion [37]. Neural crest cells contribute to aortic valve development as well as aortico-pulmonary septation [38–40]. Our previous results indicated that SLIT-ROBO signaling might be involved in regulating earlier events during cardiac neural crest cell migration that are associated to outflow tract and aortic valve development [8].

In zebrafish models, both *Slit2* and *Slit3* are expressed in the heart during chamber formation. *Slit2* is particularly expressed in endocardial cells, while *Robo1* and *Slit3* are expressed in the myocardial, endocardial and endothelial cells [7]. Slit3 is the predominant ligand transcribed in the early mouse heart. Indeed, its expression is detected in the ventral wall of the linear heart tube and subsequently in the heart chamber but not in the atrioventricular canal myocardium [8].

Functional studies using Drosophila, zebrafish, and mouse models have reported a significant role of each Robo-Slit member in heart chamber, lumen, and valve formation [3, 7, 10, 13, 14, 41]. Indeed, in *Robo1/Robo2* and *Slit3* knockout mice, the ventricular septum is absent, whereas in Slit2 mutants septum anomalies were less severe [14]. Using zebrafish models, it has been shown that *Slit3* plays a crucial role in vascular development. Similarly, in mice, *Slit3* is the earliest gene to be expressed with a strong expression in the myocardium. It is also expressed in the outflow tract, atrial and sinus horn myocardium, cardiac neural crest, the second heart field and later in the epicardium [6, 7, 13, 14]. Moreover, it has been shown that *Slit3* also still expressed in the adult ventricle [13, 14].

The phenotypic analysis of mice mutants showed that *Robo1/Robo2* mutants have developed highly penetrant BAV with two entire leaflets and one partial or absent leaflet. However *Slit2* mutants have displayed less penetrant BAV phenotype and *Slit3* mutants have thickened atrioventricular valves and hypoplastic non-coronary aortic valve [13, 14].

Additionally, it has been shown that Robo–Slit are related to the Notch and vascular endothelial growth factor signaling pathways [6, 13]. Both pathways are known to be involved in heart formation and development. Furthermore, genetic variations in *NOTCH* and *VEGF* genes have been found in patients with CHD [42, 43]. In the present study, we sought to identify genetic variants in *ROBO* and *SLIT* genes in patients with different CHD. We have identified (i) several variants with a consistent in silico prediction of pathogenicity, (ii) patients with digenic variants who have a more severe phenotype and (iii) two segregating variants, one with an autosomal recessive pattern of inheritance and one segregating with the disease in the family.

## Limitations

There may be some possible limitations in this study. The first is the limited access to detailed clinical data for the majority of patients. The second limitation concerns family segregation. Indeed, family co-segregation was possible for two cases only. Parental samples were not available for the other index-cases.

## Conclusion

Although CHD newborns are treated as soon as the disease is diagnosed, CHD persists among the most leading causes of mortality in the developed world [44]. The specific causative genetic variant remains unknown for a significant number of patients. The identification of novel variants in the *ROBO* and *SLIT* genes, as a recent associated pathway with CHD, will aid to improve the genetic testing yield of CHD. The functional effect of variants of unknown or uncertain significance remains to be elucidated as well as genotype–phenotype correlations.

Our study contributes to expand the phenotypic and allelic heterogeneity of CHD by reporting several variants in the ROBO-SLIT signaling pathway. Albeit the majority of the prioritized variants are predicted pathogenic with a consistency across different in silico predictions tools and the identification of *ROBO4* variants segregating in families, functional studies are needed to assess their clinical relevance.

## Supplementary Information

**Additional file 1. Figure S1**: Overview of ROBO1 protein in ribbon presentation. The protein is colored by element: α-helix=blue, β-strand = red, turn=green, 3/10helix=yellow, and random coil=cyan.** Figure S2**: Close-up of the* ROBO1*: p.Val610Ile variant. The protein is colored in grey, the side chain of the mutated residue is in magenta and shown as small balls. The protein is colored grey, the side chains of both the wild-type and the mutant residue are shown and colored green and red respectively** Figure S3**: Overview of* ROBO2* protein in ribbon presentation. The protein is colored by element: α-helix=blue, β-strand = red, turn=green, 3/10 helix=yellow, and random coil=cyan. Other molecules in the complex are colored grey when present.** Figure S4**: Close-up of the* ROBO2*: p.Arg811Trp variant. The protein is colored grey, and the side chains of both the wild-type and the mutant residue are shown and colored green and red respectively.** Figure S5**: Overview of* ROBO3* protein in ribbon presentation. The protein is colored by element: α-helix=blue, β-strand = red, turn=green, 3/10helix=yellow, and random coil=cyan.** Figure S6**: Close-up of the* ROBO3*: p.Thr323Met variant. The protein is colored grey, and the side chain of the mutated residue is colored magenta and shown as small balls. The side chains of both the wild-type and the mutant residue are shown and colored green and red respectively.** Figure S7**: Close-up of the* ROBO3*: p.Arg539Trp variant.** Figure S8**: Close-up of the* ROBO3*: p.Pro859Gln variant. **Figure S9**: Overview of* ROBO4* protein in ribbon presentation. The protein is colored by element: α-helix=blue, β-strand = red, turn=green, 3/10helix=yellow, and random coil=cyan.** Figure S10**: Close-up of the *ROBO4*: p.Ala303Asp variant. The protein is colored grey and the side chains of both the wild-type and the mutant residue are shown and colored green and red respectively. The side chain of the mutated residue is colored magenta and shown as small balls.** Figure S11**: Overview of* SLIT1* protein in ribbon presentation. The protein is colored by element: α-helix=blue, β-strand = red, turn=green, 3/10helix=yellow, and random coil=cyan.** Figure S12**: Close-up of the *SLIT1*: p.Pro149Leu variant. The side chain of the mutated residue is colored magenta and shown as small balls** Figure S13**: Close-up of the* SLIT1*: p.Arg455Ser variant. The side chain of the mutated residue is colored magenta and shown as small balls.** Figure S14**: Overview of* SLIT3* protein in ribbon presentation. The protein is colored by element: α-helix=blue, β-strand = red, turn=green, 3/10helix=yellow, and random coil=cyan.** Figure S15**: Close-up of the* SLIT3*: p.Ser629Asn variant. The side chain of the mutated residue is colored magenta and shown as small balls.

**Additional file 2:** Sanger sequencing of the prioritized variants.

## Data Availability

All data generated or analyzed during this study are included in this published article and its additional files.
