# Supplementary material for: Expanding the phenome and variome of the ROBO-SLIT pathway in congenital heart defects: toward improving the genetic testing yield of CHD
Source: J Transl Med. 2023 Feb 28;21:160. doi: 10.1186/s12967-023-03994-y (PMC9976407; doi:10.1186/s12967-023-03994-y)
Supplement: Supplementary file 1 — Additional file 1. Figure S1: Overview of ROBO1 protein in ribbon presentation. The protein is colored by element: α-helix=blue, β-strand = red, turn=green, 3/10helix=yellow, and random coil=cyan. Figure S2: Close-up of the ROBO1: p.Val610Ile variant. The protein is colored in grey, the side chain of the mutated residue is in magenta and shown as small balls. The protein is colored grey, the side chains of both the wild-type and the mutant residue are shown and colored green and red respectively Figure S3: Overview of ROBO2 protein in ribbon presentation. The protein is colored by element: α-helix=blue, β-strand = red, turn=green, 3/10 helix=yellow, and random coil=cyan. Other molecules in the complex are colored grey when present. Figure S4: Close-up of the ROBO2: p.Arg811Trp variant. The protein is colored grey, and the side chains of both the wild-type and the mutant residue are shown and colored green and red respectively. Figure S5: Overview of ROBO3 protein in ribbon presentation. The protein is colored by element: α-helix=blue, β-strand = red, turn=green, 3/10helix=yellow, and random coil=cyan. Figure S6: Close-up of the ROBO3: p.Thr323Met variant. The protein is colored grey, and the side chain of the mutated residue is colored magenta and shown as small balls. The side chains of both the wild-type and the mutant residue are shown and colored green and red respectively. Figure S7: Close-up of the ROBO3: p.Arg539Trp variant. Figure S8: Close-up of the ROBO3: p.Pro859Gln variant. Figure S9: Overview of ROBO4 protein in ribbon presentation. The protein is colored by element: α-helix=blue, β-strand = red, turn=green, 3/10helix=yellow, and random coil=cyan. Figure S10: Close-up of the ROBO4: p.Ala303Asp variant. The protein is colored grey and the side chains of both the wild-type and the mutant residue are shown and colored green and red respectively. The side chain of the mutated residue is colored magenta and shown as small balls. Figure S11: Overview of SLIT1 [file 12967_2023_3994_MOESM1_ESM.docx]

**Supplementary file 1:** Expanding the phenome and variome of the ROBO-SLIT pathway in Congenital Heart Defects: Toward improving the genetic testing yield of CHD; by Jaouadi et al.

This file contains the in silico prediction of the impact of the identified variant on the protein level. The tool used is a web service that analyses the structural effects of a point mutation in a protein sequence called “HOPE” (<https://www3.cmbi.umcn.nl/hope/>).

**ROBO PROTEINS AND VARIANTS**

The *ROBO1*: p.Val610Ile variant is located within the Fibronectin type-III 1 domain **(Figures S1 - S2)**. The wild-type residue is very conserved and the mutation introduces an amino acid with different properties, which can disturb this domain and abolish its function. The Fibronectin type-III 1 domain is important for binding to other molecules. The mutation might affect this interaction and thereby disturb signal transfer from the binding domain to the activity domain.


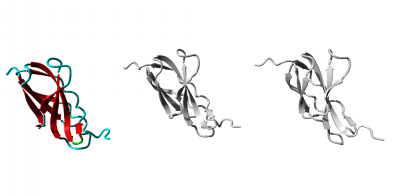


**Figure S1:** Overview of *ROBO1* protein in ribbon presentation. The protein is colored by element: α-helix=blue, β-strand = red, turn=green, 3/10helix=yellow and random coil=cyan.


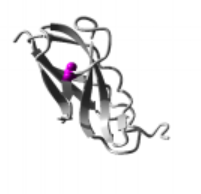

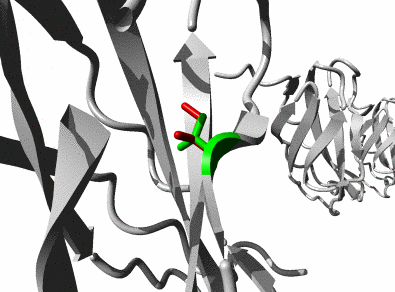


**Figure S2:** Close-up of the *ROBO1*: p.Val610Ile variant. The protein is colored in grey, the side chain of the mutated residue is in magenta and shown as small balls. The protein is colored grey, the side chains of both the wild-type and the mutant residue are shown and colored green and red respectively.


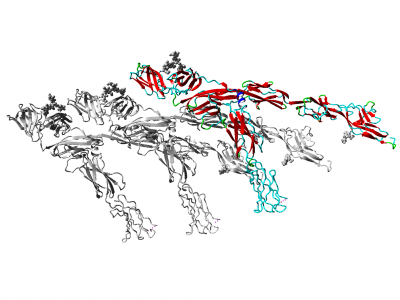


**Figure S3:** Overview of *ROBO2* protein in ribbon presentation. The protein is colored by element: α-helix=blue, β-strand = red, turn=green, 3/10 helix=yellow, and random coil=cyan. Other molecules in the complex are colored grey when present.

The *ROBO2*: p.Asp213Glu variant is located in the Ig 1 domain. The Asp residue at position 213 is very highly conserved. Thus, it is very likely to be important for the protein's function. However, the change from an Asp to a Glu side chain is not a large one, which may explain the weak in silico prediction of pathogenicity.

The *ROBO2*: p.Arg811Trp variant is located within the Fibronectin type-III 3 domain **(Figures S3 - S4)**.

The Arginine residue at position 811 forms a salt bridge with the Glutamic acid at position 813. The difference in charge will disturb the ionic interaction made by the wild-type residue.


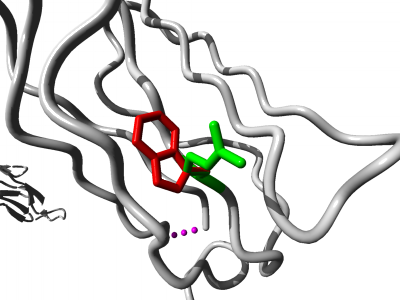


**Figure S4:** Close-up of the *ROBO2*: p.Arg811Trp variant. The protein is colored grey, and the side chains of both the wild-type and the mutant residue are shown and colored green and red respectively.


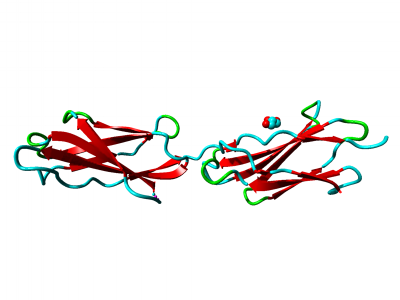


**Figure S5:** Overview of *ROBO3* protein in ribbon presentation. The protein is colored by element: α-helix=blue, β-strand = red, turn=green, 3/10helix=yellow and random coil=cyan.


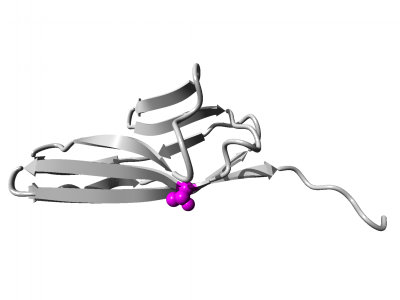

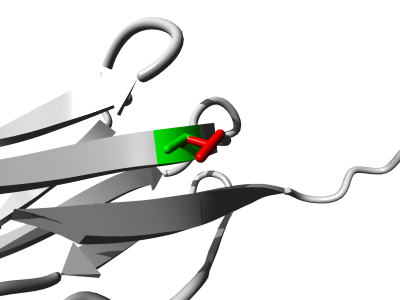


**Figure S6:** Close-up of the *ROBO3*: p.Thr323Met variant. The protein is colored grey, and the side chain of the mutated residue is colored magenta and shown as small balls. The side chains of both the wild-type and the mutant residue are shown and colored green and red respectively.

The *ROBO3*: p.Thr323Met variant is located within the Ig-like C2-type 3 domain of the gene **(Figures S5 - S6)**. The mutated residue is located on the surface of a domain with unknown function. The residue was not found to be in contact with other domains of which the function is known within the used structure. However, contact with other molecules or domains is still possible and might be affected by this mutation.


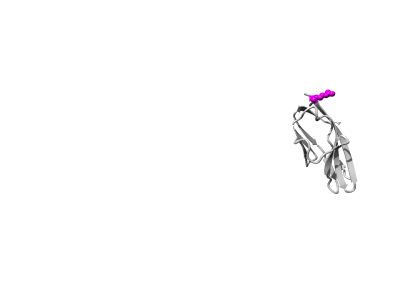

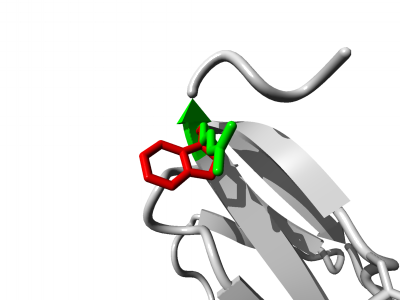


**Figure S7:** Close-up of the *ROBO3*: p.Arg539Trp variant.

The *ROBO3*: p.Arg539Trp variant: The wild-type residue (Arginine) forms a salt bridge with the Aspartic Acid at position 541, mutation of this residue can disturb interactions with other molecules or other parts of the protein **(Figure S7)**. Moreover, the difference in charge will disturb the ionic interaction made by the wild-type residue.


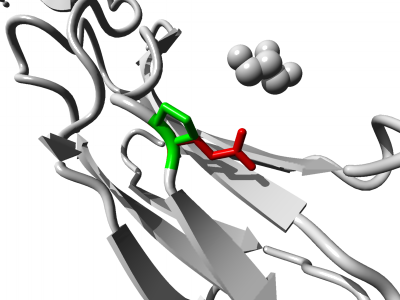


**Figure S8:** Close-up of the *ROBO3*: p.Pro859Gln variant.

The *ROBO3*: p.Pro859Gln variant is located within the Fibronectin type-III 3 domain. The mutation introduces an amino acid with different properties, which is predicted to disturb this domain and abolish its function. The wild-type residue is proline. Prolines are known to be very rigid and therefore induce a special backbone conformation that might be required at this position. The mutation can disturb this special conformation. The Pro859 residue is located on the surface of the protein, mutation of this residue can cause a loss of hydrophobic interactions with other molecules on the surface of the protein **(Figure S8)**.

The *ROBO3*: p.Gly998Val variant: The wild-type residue is glycine, the most flexible of all residues. This flexibility might be necessary for the protein's function. Mutation of this glycine can abolish this function as the torsion angles for this residue are unusual. Only glycine is flexible enough to make these torsion angles, mutation into another residue will force the local backbone into an incorrect conformation and will disturb the local structure.

The *ROBO3*: p.Pro1160Ser mutation introduces an amino acid with different properties in size and hydrophobicity. Thus, it is predicted that hydrophobic interactions, either in the core of the protein or on the surface, will be lost.


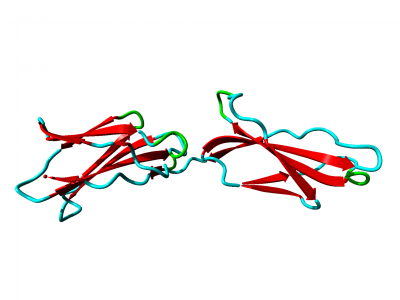


**Figure S9:** Overview of *ROBO4* protein in ribbon presentation. The protein is colored by element: α-helix=blue, β-strand = red, turn=green, 3/10helix=yellow, and random coil=cyan.


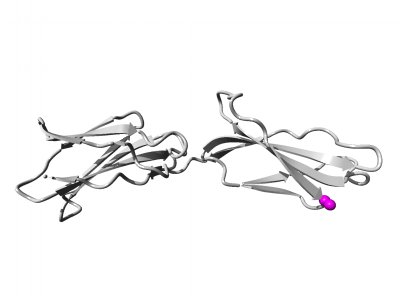

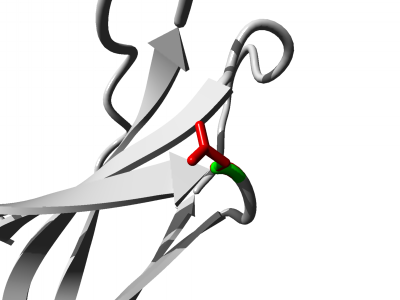


**Figure S10:** Close-up of the *ROBO4*: p.Ala303Asp variant. The protein is colored grey and the side chains of both the wild-type and the mutant residue are shown and colored green and red respectively. The side chain of the mutated residue is colored magenta and shown as small balls.

The *ROBO4*: p.Ala303Asp variant is located within the Fibronectin type-III 1 domain. The mutated residue is located on the surface of a domain that is important for the binding of other molecules **(Figures S9 - S10)**. The differences in charge and size between the wild-type and mutant residue might disturb the interaction with these other molecules.

The second *ROBO4* variant in our cohort results also in a substitution of an Alanine residue into an Aspartic acid residue (position 446). Given the fact that this substitution introduces a negative charge, this can cause the repulsion of ligands or other residues with the same charge. Furthermore, the hydrophobicity of the wild-type and mutant residue differs. Thus, hydrophobic interactions, either in the core of the protein or on the surface, will be lost.

The *ROBO4*: p.Arg776Cys variant induces differences in size, charge, and hydrophobicity. Thus, a loss of interactions with other molecules or residues can occur, and a loss of hydrogen bonds and/or disturb correct folding.

The *ROBO4*: p.Arg908Gln variant induces differences in size and charge. The mutant residue is smaller and the positive charge of the WT residue is lost, this might lead to a loss of interactions with other residues and molecules.

**SLIT PROTEINS AND VARIANTS**


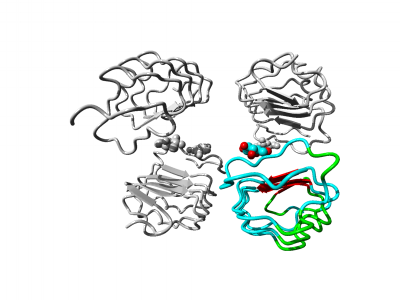


**Figure S11:** Overview of *SLIT1* protein in ribbon presentation. The protein is colored by element: α-helix=blue, β-strand = red, turn=green, 3/10helix=yellow, and random coil=cyan.

The *SLIT1*: p.Pro149Leu variant is located within the LRR 4 domain which is a stretch of residues that is repeated in the protein. The mutation into another residue might disturb this repeat and consequently any function this repeat might have. The wild-type residue is a proline (**Figures S11 – S12**). Prolines are known to be very rigid and therefore induce a special backbone conformation which might be required at this position. The mutation can disturb this special conformation.


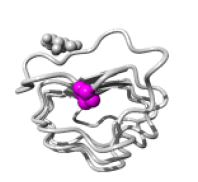


**Figure S12:** Close-up of the *SLIT1:* p.Pro149Leu variant. The side chain of the mutated residue is colored magenta and shown as small balls.

The *SLIT1*: p.Arg455Ser variant is located within the LRRCT 2 domain. The Arginine residue at position 455 forms a salt bridge with the Aspartic acid at position 452 **(Figure S13)**. The difference in charge will disturb the ionic interaction made by the wild-type residue. The alternative Serine residue is smaller than the WT residue which will cause a loss of external interactions.


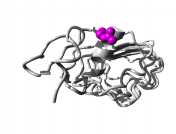

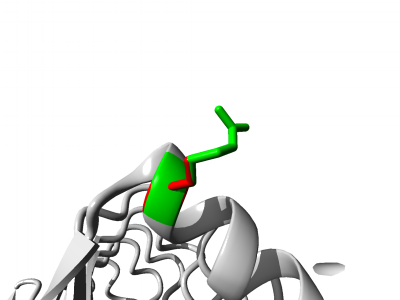


**Figure S13:** Close-up of the *SLIT1*: p.Arg455Ser variant. The side chain of the mutated residue is colored magenta and shown as small balls.

The *SLIT1*: p.Ala1253Thr variant is located within the lamin G-like domain. The mutation introduces an amino acid with different properties, which can disturb this domain and abolish its function. Hydrophobic interactions, either in the core of the protein or on the surface, will be lost.

The *SLIT1*: Glu1340Asp variant is located within the EGF-like 7 domain. The mutation introduces an amino acid with different properties, which can disturb this domain and abolish its function.

The *SLIT1*: His1382Arg variant is located within the EGF-like 8 domain. The wild-type residue is not conserved at this position. However, the mutated residue is located very close to a residue that makes a cysteine bond. This cysteine bond itself is not mutated but could be affected by the mutation located in its vicinity.

The *SLIT1*: p.Cys1401Phe variant is located within the EGF-like 8 domain. The wild-type residue is annotated in UniProt to be involved in a cysteine bridge, which is important for the stability of the protein. Only cysteines can make these types of bonds, the mutation causes loss of this interaction and will have a severe effect on the 3D-structure of the protein. Together with the loss of the cysteine bond, the differences between the WT and new residue can cause destabilization of the structure.

The *SLIT2*: p.Leu1293Met variant is located within the lamin G-like domain. The mutation introduces an amino acid with different properties, which can disturb this domain and abolish its function.

The *SLIT3*: p.Arg494Thr variant induces differences in size, charge, and hydrophobicity. Thus, a loss of interactions with other molecules or residues can occur, and a loss of hydrogen bonds and/or disturb correct folding.


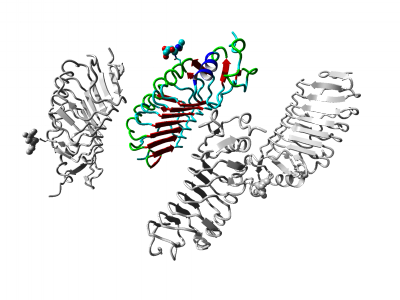


**Figure S14:** Overview of *SLIT3* protein in ribbon presentation. The protein is colored by element: α-helix=blue, β-strand = red, turn=green, 3/10helix=yellow, and random coil=cyan.

The *SLIT3*: p.Ser629Asn variant is located in a Leucine-Rich Repeat domain that is important for the binding of other molecules. The mutated residue is in contact with residues in another domain. It is possible that the mutation disturbs these contacts **(Figures S14 - S15)**.


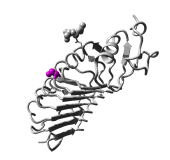

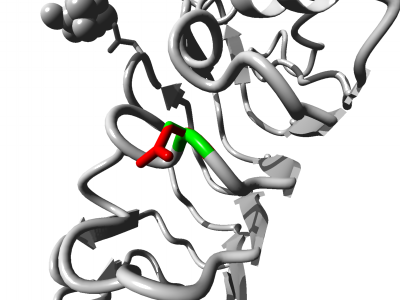


**Figure S15:** Close-up of the *SLIT3*: p.Ser629Asn variant. The side chain of the mutated residue is colored magenta and shown as small balls.
