# Supplementary material for: Expanding the phenome and variome of the ROBO-SLIT pathway in congenital heart defects: toward improving the genetic testing yield of CHD
Source: J Transl Med. 2023 Feb 28;21:160. doi: 10.1186/s12967-023-03994-y (PMC9976407; doi:10.1186/s12967-023-03994-y)
Supplement: Supplementary file 2 — Additional file 2: Sanger sequencing of the prioritized variants. [file 12967_2023_3994_MOESM2_ESM.docx]

**Supplementary file 2:** Expanding the phenome and variome of the ROBO-SLIT pathway in Congenital Heart Defects: Toward improving the genetic testing yield of CHD; by Jaouadi et al.

Sanger sequencing of the prioritized variants.


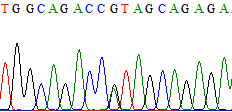


*ROBO1*: c.1828G>A: p.Val610Ile (Patient ID: BAV-AD-1)


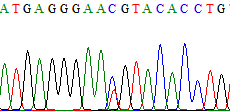


*ROBO3*: c.968C>T: p.Thr323Met (Patient ID: TOF-PED-2)


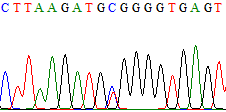


*ROBO3*: c.1615C>T: p.Arg539Trp (Patient ID: TOF-PED-3)


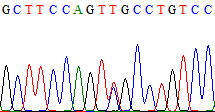


*ROBO4*: c.2326C>T: p.Arg776Cys (Patient ID: TOF-PED-9)


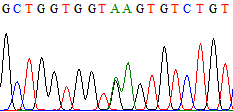


*ROBO4*: c.3001+3G>A (Patient ID: TOF-PED-11)


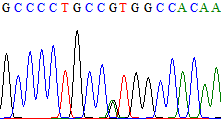


*SLIT1*: c.4145A>G: p.His1382Arg (Patient ID: BAV-AD-3)


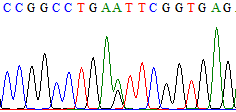


*SLIT3*: c.1886G>A : p.Ser629Asn (Patient ID: BAV-AD-4)
